# Construction of a Diagnostic Model for Small Cell Lung Cancer Combining Metabolomics and Integrated Machine Learning

**DOI:** 10.1093/oncolo/oyad261

**Published:** 2023-09-14

**Authors:** Xiaoling Shang, Chenyue Zhang, Ronghua Kong, Chenglong Zhao, Haiyong Wang

**Affiliations:** Department of Clinical Laboratory, Shandong Cancer Hospital and Institute, Shandong University, Jinan, People’s Republic of China; Department of Integrated Therapy, Fudan University Shanghai Cancer Center, Shanghai Medical College, Shanghai, People’s Republic of China; Department of Breast Surgery, Shandong Cancer Hospital and Institute, Shandong First Medical University and Shandong Academy of Medical Sciences, Jinan, People’s Republic of China; Department of Pathology, The First Affiliated Hospital of Shandong First Medical University and Shandong Provincial Qianfoshan Hospital, Jinan, Shandong, People’s Republic of China; Department of Internal Medicine-Oncology, Shandong Cancer Hospital and Institute, Shandong First Medical University and Shandong Academy of Medical Sciences, Jinan, People’s Republic of China

**Keywords:** SCLC, diagnosis model, metabolomics, lipidomics, machine learning

## Abstract

**Background:**

To date, no study has systematically explored the potential role of serum metabolites and lipids in the diagnosis of small cell lung cancer (SCLC). Therefore, we aimed to conduct a case-cohort study that included 191 cases of SCLC, 91 patients with lung adenocarcinoma, 82 patients with squamous cell carcinoma, and 97 healthy controls.

**Methods:**

Metabolomics and lipidomics were applied to analyze different metabolites and lipids in the serum of these patients. The SCLC diagnosis model (d-model) was constructed using an integrated machine learning technology and a training cohort (*n* = 323) and was validated in a testing cohort (*n*=138).

**Results:**

Eight metabolites, including 1-mristoyl-sn-glycero-3-phosphocholine, 16b-hydroxyestradiol, 3-phosphoserine, cholesteryl sulfate, D-lyxose, dioctyl phthalate, DL-lactate and Leu-Phe, were successfully selected to distinguish SCLC from controls. The d-model was constructed based on these 8 metabolites and showed improved diagnostic performance for SCLC, with the area under curve (AUC) of 0.933 in the training cohort and 0.922 in the testing cohort. Importantly, the d-model still had an excellent diagnostic performance after adjusting the stage and related clinical variables and, combined with the progastrin-releasing peptide (ProGRP), showed the best diagnostic performance with 0.975 of AUC for limited-stage patients.

**Conclusion:**

This study is the first to analyze the difference between metabolomics and lipidomics and to construct a d-model to detect SCLC using integrated machine learning. This study may be of great significance for the screening and early diagnosis of SCLC patients.

## Teaser

A novel and solid diagnostic model has been developed for small cell lung cancer (SCLC) diagnosis. Its incorporation into a clinician’s arsenal could significantly improve the ability to identify patients with SCLC and even better if combined with the conventional markers NSE and ProGRP.

**Implications for Practice**

The authors have developed a novel and solid diagnostic model for small cell lung cancer (SCLC) diagnosis. The 8-metabolite diagnostic panel offers a feasible and convenient strategy for the diagnosis of SCLC. The metabolic panel has an excellent diagnostic value, which is informative and meaningful to physicians and can be quickly adopted in clinical practice. Its incorporation into a clinician’s arsenal could significantly improve the ability to identify patients with SCLC and even better if combined with the conventional markers NSE and ProGRP.

## Introduction

Small cell lung cancer (SCLC) accounts for approximately 15% of all lung cancers. SCLC is an aggressive high-grade neuroendocrine malignant tumor characterized by rapid doubling time, an early tendency to widespread metastasis, and extremely poor survival.^1^ Most patients develop distant metastasis at the time of initial diagnosis, resulting in a median survival duration of <1 year. Importantly, if treatment is not given, the median survival time is only 3-4 months.^1,2^ Therefore, early and effective detection of SCLC will allow timely treatment and positively impact the prognosis.

Neuron-specific enolase (NSE) and ProGRP are currently used as tumor biomarkers for SCLC diagnosis, but they have poor sensitivity and specificity to predict SCLC disease.^3-11^ Although the predictive ability of ProGRP is higher than that of NSE, the diagnostic sensitivity of ProGRP varied within the range of 54%-78%, with diagnostic specificity of 72%-99%.^12^ In addition, other non-neuroendocrine makers, such as caspase cleaved cytokeratin 19 (CYFRA21-1) and lactate dehydrogenase (LDH), are commonly used to diagnose SCLC. However, the area under the receiver-operating characteristic (ROC) curves (AUC) of CYFRA21-1 and LDH was reported to be 0.732 and 0.616, respectively, in SCLC diagnosis.^13^ In recent years, an increasing number of studies have demonstrated that blood-based tumor components such as circulating tumor DNA (ctDNA), circulating tumor cells (CTCs), exosomes, and extracellular vesicles (EVs) could provide an opportunity to assess biomarkers for SCLC diagnosis.^14-17^ However, most tumor markers are not suitable for screening purposes and histological diagnosis when applied separately, each one having its own limitations.^13^ For instance, analysis of cell-free DNA (cfDNA), have emerged as a promising noninvasive diagnostic approach in tumor. Analysis of cfDNA methylation profiles has been used for the early detection of cancer. Some component of the overall cfDNA is released by tumor cells, as termed ctDNA. There have been many studies reporting the utility of ctDNA methylation in the early detection of cancer. Despite great interest in cfDNA as an approach for the early detection of cancer, it has the following demerits: (1) minimal cfDNA cannot be detected sensitively in tumors harboring low tumor shedding rate,^18^ (2) ctDNA may be subject to degradation as its unstable property,^19^ (3) disaccord may exist due to ctDNA methylation signatures identified by different bioinformatics model, which could result in bias in the model,^20^ and (4) additionally, these biomarkers are largely dependent on the methylation level of CpG sites and the sensitivity can be greatly affected by technical issues.^21^

Metabolic enhancement is one of the hallmarks of cancer. Metabolomics and lipidomics are “omics” technologies that can provide additional profiling information. More importantly, metabolites and lipids are quantifiable in body fluids, allowing for non-invasive disease diagnosis.^22^ Many studies have shown that metabolite biomarker panels exhibited good diagnostic performance for detecting cancers.^23-25^ However, the potential role of serum metabolites and lipids in the diagnosis of SCLC patients has not been systematically explored.

In this study, we used liquid chromatography-tandem mass spectrometry (LC-MS/MS) to perform metabolomics and lipidomics on serum samples collected from 461 subjects, including SCLC, non-small cell lung cancer (NSCLC), and healthy participants to establish a metabolite/lipid-based model for the detection of SCLC.

## Materials and Methods

### Sample Collection

A total of 501 serum samples were collected from Shandong Cancer Hospital and Institute from March to November 2020. After excluding samples with unclear medication information and substandard serum quality, a total of 461 participants, including 191 cases of SCLC, 173 cases of NSCLC, and 97 healthy controls, were enrolled in our study. Most of the patients with SCLC were under 65 years old, and the proportion of males was 70.7%. Among the patients with NSCLC, there were 91 patients with lung adenocarcinoma and 82 patients with squamous cell carcinoma. The detailed clinical characteristics are shown in Supplementary Table S1.

The status of all patients with cancer was confirmed by histopathology, and blood samples were collected before any anti-tumor treatment. All blood samples were centrifuged at 3000 × *g* for 10 minutes no later than 6 h after sample collection to obtain serum samples, and serums were stored at −80 °C until use.

### Extraction of Metabolites and Lipids

Metabolite extraction and lipid extraction can be seen in Supplementary Materials and Methods.

### Quality Evaluation of Experimental Data

Evaluation of untargeted metabolomics and lipidomics data is shown in Supplementary Materials and Methods.

### LC-MS/MS Analysis

A detailed analysis of untargeted metabolomics and lipidomics is shown in Supplementary Materials and Methods.

### Difference Analysis of Metabolites and Lipids

Metabolite and lipid profiles from 461 cases were randomly distributed in training (*n* = 323) and testing cohorts (*n* = 138). In the training cohort, the principal component analysis (PCA) method was used to observe the overall distribution trend and the degree of difference of the samples between groups. Partial least squares discrimination analysis (PLS-DA) is a supervised statistical method of discriminant analysis. The differential lipids related to grouping can be excluded from the data set through the established discriminant model. orthogonal projections to latent structures discriminant analysis (OPLS-DA) is a modified analysis method for PLS-DA, which can filter out the noise independent of classification information and improve the analytical ability and effectiveness of the model. The variable importance in projection (VIP) obtained from the OPLS-DA model can be used to measure the influence of the expression patterns of metabolites and lipids on the classification of each group of samples and the explanatory ability to mine biologically significant differential lipid molecules. Metabolites/lipids with VIP > 1 are considered to have a significant contribution to model interpretation. Metabolomics and lipidomics take OPLS-DA VIP > 1 and *P* < .05 as the screening criteria for significant differences in metabolites in our study. When analyzing the differences between the 2 groups of samples, commonly used univariate statistical analysis methods include FC analysis, *T*-test, and non-parametric test. Based on univariate analysis, the differences of all metabolites and lipids were analyzed. Differential metabolites and lipids of FC > 1.5 or FC < 0.67, *P* < .05 were visually displayed in a volcano map. Importantly, PCA, PLS-DA, and OPLS-DA were obtained by 7-fold cross-validation in our study. Model evaluation parameters including *R*^2^ and *Q*^2^ were used to evaluate the stability and reliability of the model. A permutation test was applied to ensure the validity of the model.

### Functional Enrichment Analysis

Correlation analysis was applied to measure the metabolic closeness between metabolites with significant differences (VIP > 1, *P* < .05) to better understand the mutual regulation of metabolites in the process of biological state changes. In organisms, different metabolites coordinate with each other to exercise their biological functions. Correlated expression of metabolites might suggest joint participation in a biological process; it could also indicate a synergistic or mutually exclusive relationship between different metabolites. The positive correlation of the metabolites could also indicate that they originate from the same synthetic pathway, while the negative correlation could indicate that they might be decomposed to synthesize other metabolites. Kyoto Encyclopedia of Genes and Genomes (KEGG) pathway enrichment analysis (http://www.kegg.jp/) for significant differential metabolites (VIP > 1, *P* < .05) was used to analyze and calculate the significant level of enrichment of metabolites in each pathway and to determine the metabolic and signal transduction pathways using Fisher’s exact test.

### Construction of the Diagnostic Model

**Figure 1.**
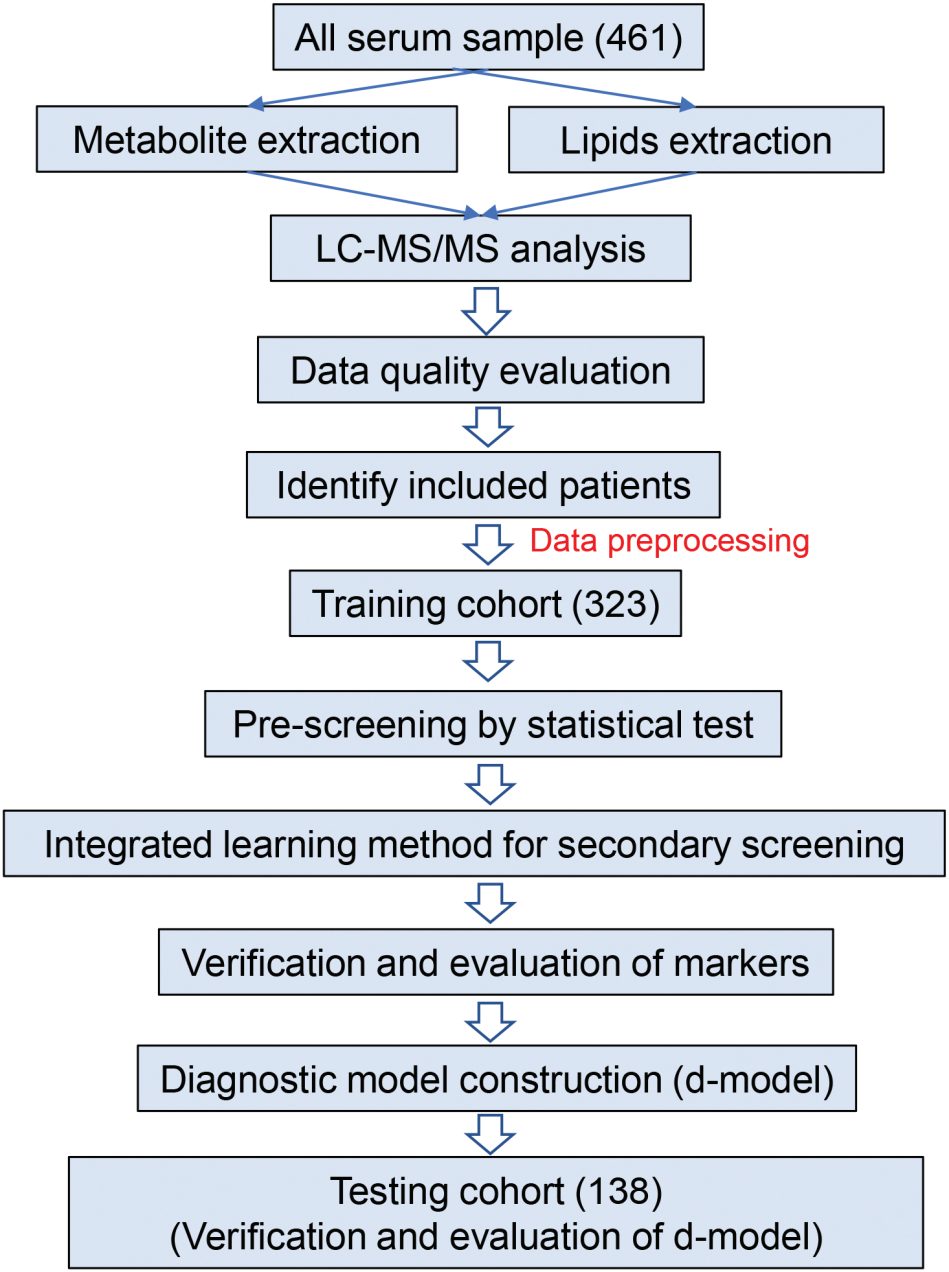
The flow chart to construct diagnostic model of SCLC.

The process of constructing the diagnostic model is presented in Fig. 1. Specifically, all serum samples were used to extract metabolites and lipids. After liquid chromatography-tandem mass spectrometry LC-MS/MS analysis, the data quality were evaluated, and stable and reliable data were further analyzed to identify included patients. Then, pre-screening by statistical test and the integrated learning method were applied to a training cohort of 66 healthy subjects and 123 NSCLC patients, and 134 SCLC patients, leading to a final selection of candidate markers by the weight value. In addition, to effectively screen candidate biomarkers, ROC analysis was used to evaluate the influence intensity of each substance on the AUC value of the model. Three machine learning models, including logistic regression (LR), random forest (RF), and support vector machine (SVM), were used to verify the candidate biomarkers. The performance of candidate markers for different groups was determined by ROC analysis. Using the classification model constructed by the RF algorithm, the importance coefficient of the candidate biomarkers was calculated and used to compare the contribution of each biomarker to the model. Then, a panel of biomarkers for diagnosis was constructed using a logical regression algorithm. The logical regression coefficient and the intercept of each candidate marker were calculated as follows: $p=\frac{1}{1+e^{-z}}$, *z* = intercept + coefficient (B1) + coefficient (B2) + coefficient (B3) +··· + coefficient (Bn). The expression level of biomarkers was brought into the calculation of the above panel model formula through Z score conversion. The successfully constructed model was named “d-model” in our study. In order to obtain this boundary value, we used Youden’s index to define the best cutoff value the diagnostic decision. Youden’s index defined the best cutoff value of diagnostic decisions and has been widely used.^26-28^ When the same weight has been given to sensitivity and specificity, the cutoff of maximum Youden’s index is an optimal value to differentiate biomarkers. Based on the expression of candidate biomarkers, the constructed d-model was evaluated by ROC curve analysis. The calculated d-model score, if the score exceeds the cutoff value, was judged to be positive for diagnosis. Finally, the diagnostic model was verified by ROC curve analysis for the testing cohort (healthy = 31, NSCLC = 50, SCLC = 57).

### Statistical Analysis

We have used *t*-test for the comparison between the 2 groups. All statistical analyses were 2 sided, and a *P*-value <.05 was considered statistically significant. The processed data were subjected to multivariate analysis in R packages (R version 4.1.3). All related instruments and reagent material information are presented in Supplementary Material 1. All the software and platforms used for statistical analysis are presented in Supplementary Material 2.

## Results

### Difference of Metabolites and Lipids

**Figure 2.**
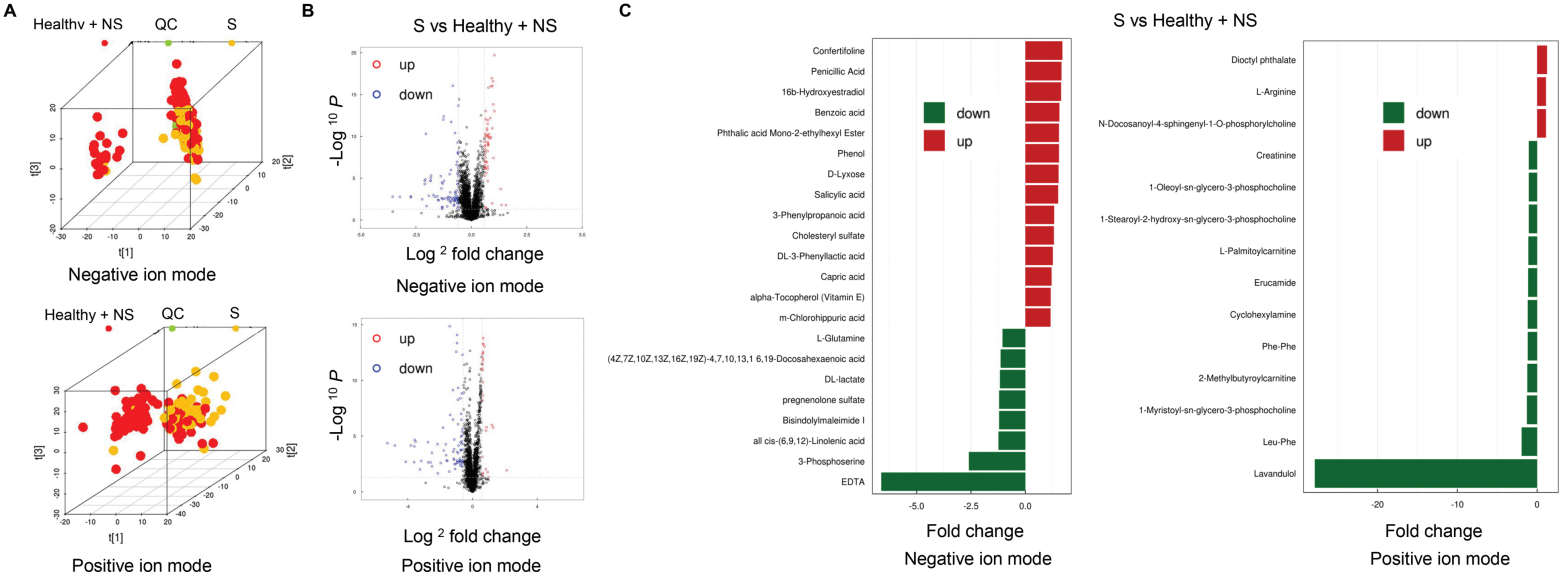
The metabolites difference between the SCLC and control (NSCLC+healthy) in the training cohort. **A**: Three-dimensional PCA score plot of the metabolites difference. **B**: The volcano plots of the metabolites difference. **C**: The specific metabolites with lower levels and elevated levels in negative/positive ionization mode.

A 3-dimensional PCA score plot showed that the metabolites and lipid profiles of SCLC patients generally differed from the control (NSCLC + healthy) (Fig. 2A; Supplementary Figs. S1, S2, S3A; and Tables S2–S4). Next, PLS-DA and OPLS-DA were performed to maximize variations between different groups and determine the metabolites and lipids that contributed discriminant to this variation. The PLS-DA (negative ionization mode: *Q*^2^ = 0.958; positive ionization mode: *Q*^2^ = 0.885) and OPLS-DA (negative ionization mode: *Q*^2^ = 0.935; positive ionization mode: *Q*^2^ = 0.905) scores plot illustrated a clear separation of metabolites between SCLC and control (NSCLC + healthy; Supplementary Fig. S4). However, the separation of lipids was not apparent (Supplementary Fig. S3C–D). Next, volcano plots were used to visualize the metabolites and lipids with *P* < .05 and FC > 1.5 or < .67. The results showed more differences in metabolites than lipids (Fig. 2B; Supplementary Fig. 3B; Supplementary Tables S2–S4). In addition, the 14 metabolites with elevated levels and 8 metabolites with low levels were identified in negative ionization mode (Fig. 2C). Furthermore, the 3 metabolites with elevated levels and 11 metabolites with low levels were identified in positive ionization mode (Fig. 2C). Furthermore, the significant differential lipid molecules (VIP > 1, *P* < .05) selected in this study were visualized in the form of a bubble diagram (Supplementary Fig. S3E; Supplementary Table S5). In addition, there was no obvious correlation between these lipids (Supplementary Fig. S3F).

### Screening Biomarkers Using Machine Learning

**Figure 3.**
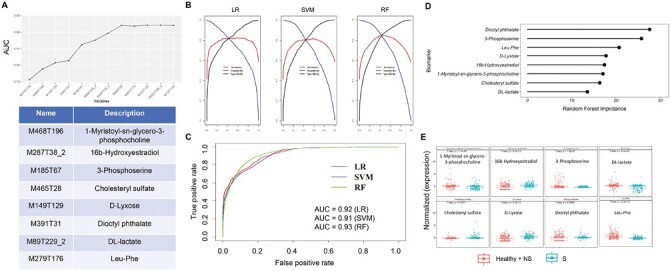
Definite diagnostic biomarkers of SCLC. **A**: The AUC accumulation curve for screened 8 metabolites. **B**: Three machine learning methods including LR, RF, and SVM were used to definite the 8 metabolites. **C**: The ROC curve of definite biomarker using LR, RF, and SVM. **D**: The random forest importance of 8 metabolites. **E**: The normalized expression of 8 metabolites between the SCLC and control (NSCLC + healthy).

Differentially expressed metabolites and lipids were analyzed twice using the ensemble learning method, and the weight value of each substance was calculated. The results showed that the weight of metabolites was much greater than that of lipids (Supplementary Fig. S5). To effectively select candidate biomarkers, ROC analysis was used to evaluate the influence intensity of each substance on the AUC value of the model. The AUC accumulation curve showed that the substances with the top 8 weights could improve the sample classification ability, including 1-Mristoyl-sn-glycero-3-phosphocholine, 16b-hydroxyestradiol, 3-phosphoserine, cholesteryl sulfate, D-lyxose, dioctyl phthalate, DL-lactate and Leu-Phe (Fig. 3A). Subsequently, 3 machine learning methods, namely LR, RF, and SVM, were used to verify the screened results, and the performance of 8 candidate metabolites in the classification of the SCLC group and control group samples were analyzed by the ROC curve (Fig. 3B). In our study, we have used 3 ML algorithms (RF, LR, and SVM) to achieve the optimal combination of the diagnostic model. The results revealed that the AUC of the LR model was 0.92, the AUC of the SVM model was 0.91, and the AUC of the RF model was 0.93 (Fig. 3C). In addition, the importance coefficients of 8 metabolites were calculated using the classification model constructed by a LR algorithm to compare the contribution of each metabolite to the model. The results showed that dioctyl phthalate contributed the most to the model, while DL-lactate contributed the least (Fig. 3D). Moreover, the expression levels of 8 metabolites in the SCLC group and control group were analyzed. The results revealed that 1-mristoyl-sn-glycero-3-phosphocholine (*P* = 1.1e−05), 16b-hydroxyestradiol (*P* = 2.7e−12), 3-phosphoserine (*P* = 1.6e−07), DL-lactate (*P* = 4.4e−09), cholesteryl sulfate (*P* = .00011), d-lyxose (*P* = 5.3e−12), dioctyl phthalate (*P* = .0069) and Leu-Phe (*P* < 2.2e−16) had significant differences in 2 groups (Fig. 3E). The Pearson correlation coefficient was calculated for the expression of the 8 metabolites, which showed that the correlation of the 8 candidate biomarkers was low (Supplementary Fig. S6). In addition, the KEGG pathway enrichment analysis demonstrated that the 8 metabolites were mainly enriched in HIF-1 signaling pathway, pyruvate metabolism, cAMP signaling pathway, glucagon signaling pathway, glycolysis/gluconeogenesis, central carbon metabolism in cancer, propanoate metabolism, glycine, serine, and threonine metabolism and aminoacyl-tRNA biosynthesis (Supplementary Fig. S7A). Supplementary Fig. S7B revealed that the 8 metabolites were enriched in pyruvate metabolism.

### Construction of d-Model

**Figure 4.**
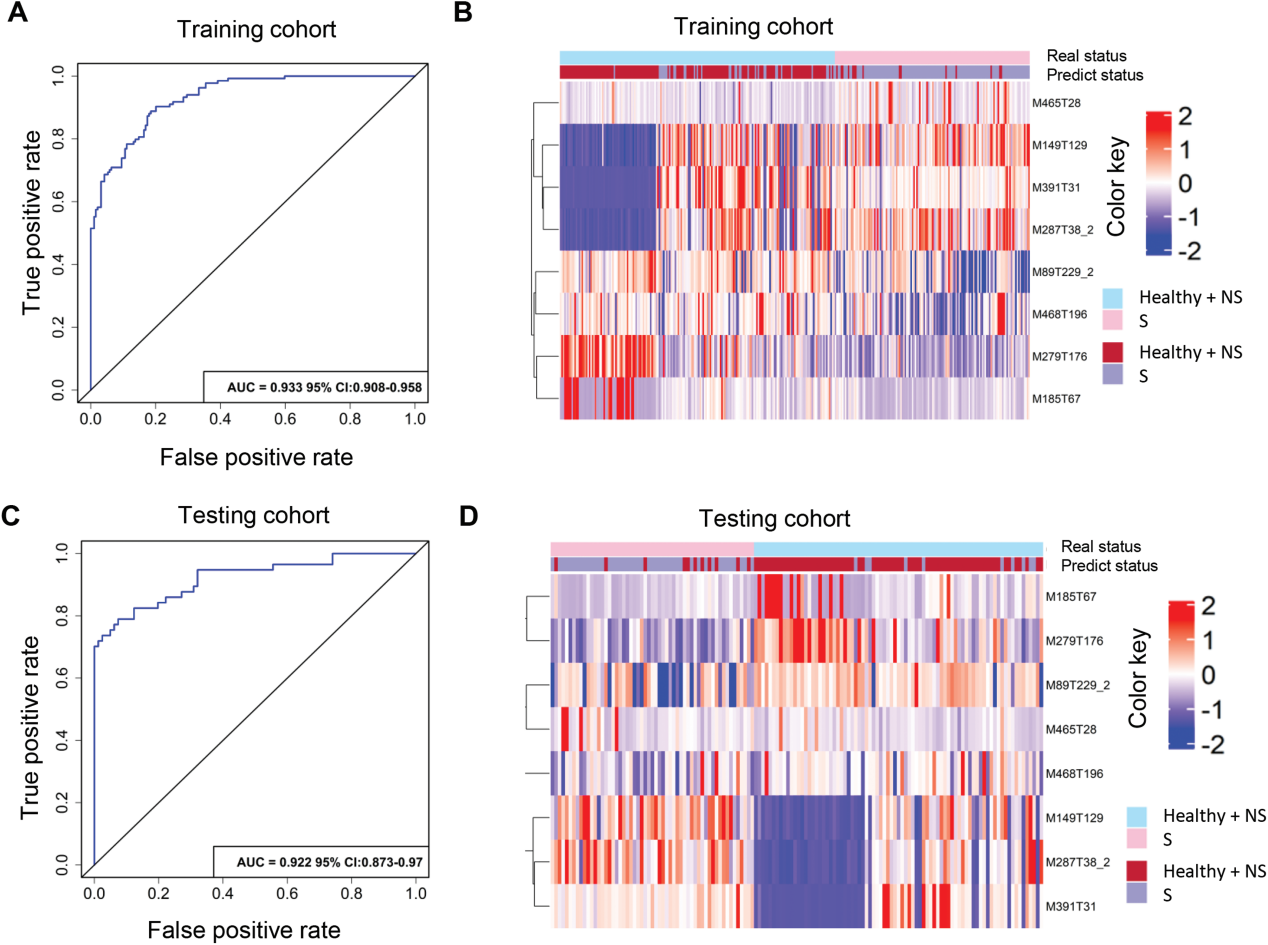
Construction and validation of d-model in SCLC. **A**: The ROC curve in the training cohort. **B**: The clustering heat map in the training cohort. **C**: The ROC curve in the testing cohort. **D**: The clustering heat map in the testing cohort.

Next, the samples of the training cohort were used to construct d-model for SCLC. The result showed that the AUC of the model was 0.933 (95% CI: 0.908-0.958; Fig. 4A). The clustering analysis of the 8 metabolites in the training cohort showed a clear separation between 2 groups in the heatmap (Fig. 4B). Then, the rest samples of testing cohort were used to verify the applicability of the d-model. The result revealed that the AUC of the model in the testing cohort was 0.922 (95% CI: 0.873-0.97; Fig. 4C). Moreover, the clustering analysis of the 8 metabolites in the testing cohort revealed a clear separation between the 2 groups (Fig. 4D).

### The d-Model Could Improve the Diagnostic Performance for SCLC

**Figure 5.**
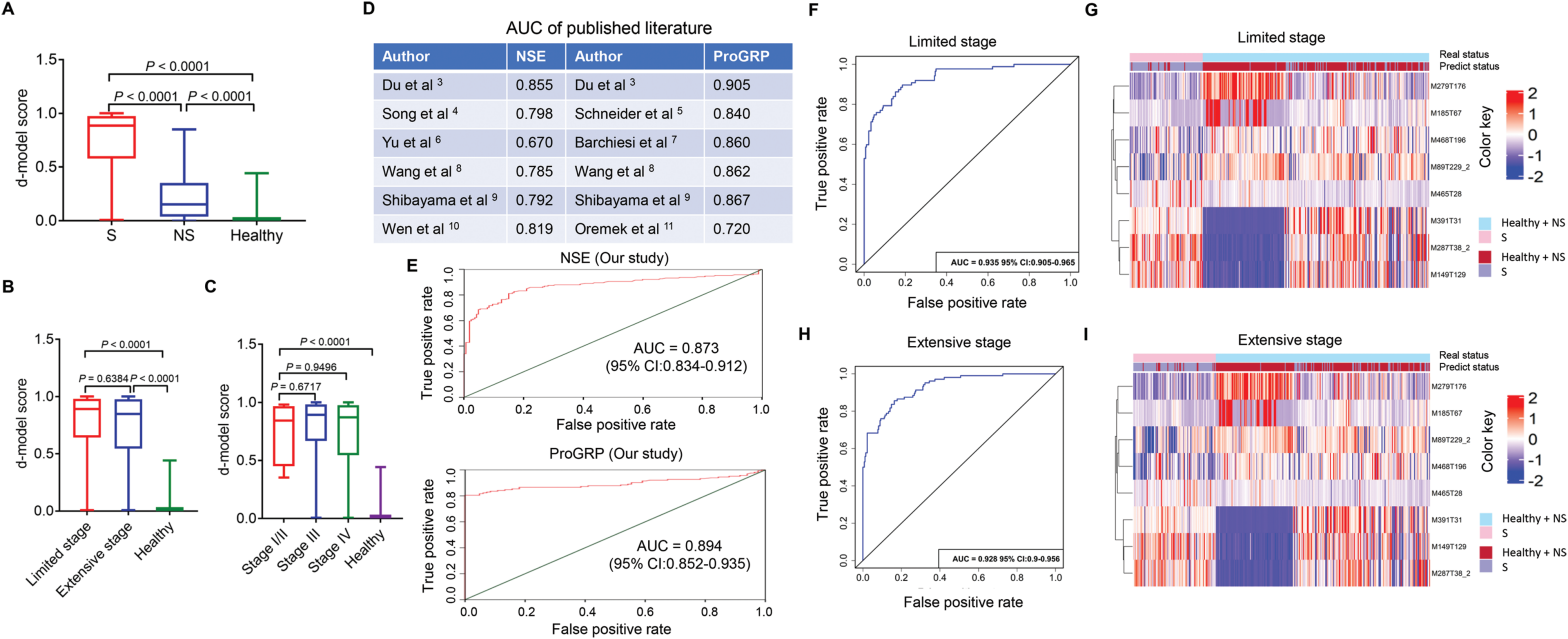
The diagnostic performance of SCLC-based on different subgroups. **A**: The d-model score difference between SCLC and control. **B** and **C**: The d-model score difference of SCLC with different stage. **D**: Published AUC of NSE and ProGRP in SCLC. **E**: The ROC curve of NSE and ProGRP in our study. **F**: The ROC curve for limited-stage SCLC. **G**: The clustering heat map for limited-stage SCLC. **H**: The clustering heat map for extensive-stage SCLC. **I**: The clustering heat map for extensive-stage SCLC.

Finally, all 461 samples were used to assess the diagnostic performance of the d-model for SCLC. We found that SCLC exhibited a high d-model score compared to NSCLC (*P* < .0001) and healthy controls (*P* < .0001; Fig. 5A). Having the same diagnostic effect at different stages is an important value for any diagnostic biomarker. There is no significant difference in the d-model score, whether it is the Veterans Administration Lung Study Group (VALG) staging or the American Joint Committee on Cancer (AJCC) staging (Fig. 5B–5C), suggesting that the diagnostic performance of the signature was independent of tumor burden and would make it an optimal diagnostic tool for the detection of SCLC. Previous studies reported that the AUC of NSE for SCLC ranged from 0.670 to 0.850, and that of ProGRP for SCLC ranged from 0.720 to 0.905 (Fig. 5D). Moreover, we analyzed the diagnostic efficacy of NSE and ProGRP in our study. The result showed that the AUC of NSE and ProGRP for the detection of SCLC was 0.873 and 0.894, respectively (Fig. 5E). In addition, we confirmed the diagnostic performance of the d-model in SCLC of different staging. The AUC for patients with limited-stage and extensive stage cancers were 0.935 and 0.928, respectively (Fig. 5F and H). The AUCs for patients with stages I/II, III, and IV cancers compared with controls were 0.904, 0.939, and 0.927, respectively (Supplementary Fig. S8A, S8C, and S8E). Unsupervised hierarchical clustering using the 8 metabolites effectively distinguished different stages of SCLC from controls with high specificity and sensitivity (Fig. 5G and 5I; Supplementary Fig. S8B, S8D, and S8F). Importantly, the d-model still shows a good diagnostic ability after adjusting age (AUC: 0.935 for <65; 0.925 for ≥65), gender (AUC: 0.917 for female; 0.937 for male), smoking history (AUC: 0.937 for yes; 0.922 for no), drinking history (AUC: 0.924 for yes; 0.934 for no), and medication history (AUC: 0.926 for yes; 0.933 for no; Supplementary Table S6). These results show that the d-model is dominant and stable in SCLC detection. Interestingly, we also found that this d-model combined with NSE (AUC: 0.952) or ProGRP (AUC: 0.962) showed better diagnostic performance to detect SCLC (Supplementary Fig. S9A and S9B). Another great value of diagnostic markers is early detection. Since only 10 patients (5%) with SCLC were in stage I/II (AJCC) in our study, we selected limited-stage patients with SCLC to verify the early diagnostic performance of the combined diagnostic model. The results that showed d-model combined with NSE of limited-stage patients had a relatively good diagnostic performance with 0.961 of AUC compared with patients with NSCLC (Supplementary Fig. S9C). Importantly, the d-model combined with ProGRP of limited-stage patients showed the best diagnostic performance with 0.975 of AUC compared with patients with NSCLC (Supplementary Fig. S9D).

## Discussion

SCLC is a highly lethal disease that demonstrates many malignant features.^29,30^ Featured by the metastatic nature and the lack of effective treatments,^1^ early screening and detection of SCLC is vital to improving patient survival and quality of life. However, even though CT screenings have been performed among high-risk patients, we still fail to detect early-stage SCLC to find most patients at stage IV upon diagnosis.^31^ Therefore, there is an urgent unmet need for new approaches in the detection of SCLC.

The exploration of biomarkers for the detection of SCLC has been a pursuit of researchers. Conventional biomarkers such as NSE and ProGRP are elevated in some SCLC cases, but their specificity and sensitivity are insufficient for an accurate diagnosis.^32,33^ Although some proteins, microRNAs, and exosomes have been subsequently adapted to detect SCLC,^17,34,35^ specific biomarkers capable of identifying SCLC with relatively high specificity and sensitivity have not yet been discovered. Moreover, a single biomarker is insufficient for a solid and convincing diagnosis. Therefore, identification of a panel of potential biomarkers would be of clinical relevance for SCLC diagnosis.

Tumor development and progression are often accompanied by metabolic changes. The metabolism of cancer cells is rewired to offer sufficient energy for cell proliferation to survive in a hypoxic and nutrient-deprived tumor microenvironment. In particular, metabolomics could even detect the most subtle and minor changes in circumstances where no measurable genetic or protein changes are detected.^36-40^ Therefore, metabolites may serve as suitable candidates as cancer biomarkers. Additionally, dysregulation of lipid metabolism has been reported to be associated with cancer. For lipid disorders in cancer, numerous targets have also been discovered as therapeutic interventions.^41-43^ Therefore, in the present study, we conducted both metabolomic and lipidomic analyses to discover biomarkers for an improved diagnosis of SCLC.

To optimize the screening of biomarkers, we performed machine learning of serum metabolites and lipids for diagnosis of SCLC, the approach of which could enhance the accuracy of our screened biomarkers and abate the dimensionality of data sets.^44,45^ Notably, a 7-fold cross-validation approach was applied to identify optimized biomarkers on the serum samples in the study. The merit of this approach lies in that it can avoid information leakage and mitigate underfitting and overfitting during each step.^46^

Interestingly, a biomarker panel consisting of 8 metabolites, whereas no lipids have been demonstrated in SCLC patients versus NSCLC patients and healthy participants in the present study. Previous studies have reported that lipids play a crucial role in cancer cells undergoing progression and metastasis. Lipid metabolism perturbation is considered a hallmark of cancer, and many researchers have centered on altered lipids in cancers.^47,48^ For example, saturated fatty acids are altered in colorectal cancer.^49^ Furthermore, phospholipids were found to be differentiated between lung cancer patients and healthy participants.^50^ In a study, lipid-based serum signature demonstrated potential utility in discriminating early patients with lung cancer from healthy controls (HC).^51^ Another study led by Chen, it has found a significantly altered lipid metabolic profile in early-stage NSCLC and identified panels of phosphatidylcholines and phosphatidylethanolamines to distinguish NSCLC patients and HC.^52^ In contrast, the aim of our study is to find a biomarker to differentiate between SCLC and HC, as well as to distinguish between SCLC and NSCLC. In our present study, it is found that the difference in lipids between SCLC and NSCLC is relatively small and that lipids are not a promising biomarker for SCLC diagnosis. Previous metabolomics studies have been devoted to explore reliable biomarkers using plasma, serum, or urine for screening and diagnosing lung cancer. Kim J et al demonstrated the preliminary feasibility of distinguishing early-stage NSCLC cases from controls and adenocarcinomas from squamous cell carcinomas using metabolomics techniques.^53^ However, the authors did not include cases of SCLC patients. The study led by Lee S et al included 27 patients with SCLC and identified association between metabolites and initial status and outcomes of SCLC.^54^ Furthermore, Shi et al demonstrated that using 5+ metabolites has the potential for early lung cancer screening.^55^ However, previous studies may have deviations due to the small sample size. In the contrast to previous study, we included 461 subjects, including 191 SCLC, 173 NSCLC, and 97 healthy participants to perform metabolomics and lipidomics and successfully established a diagnostic model based on 8 metabolites for the detection of SCLC. It has to be noted that, we have used 3 ML algorithms (RF, LR, and SVM) to achieve the optimal combination of the diagnostic model in our present study. Data from logic regression algorithm and the optimized potential biomarker have been used for the construction of diagnostic model. LR is a both simple and explicable model. Based on the LR model, the weight coefficient, a parameter that reflects the contribution to the model, can be directly manifested. A quadratic integrated machine learning algorithm is used for the screening of high-performance features, as well as the output of LR model construction. Therefore, an LR model-based score was used for clinical diagnosis in our study.

The metabolites identified in our study contain several known compounds that have already been reported, such as 3-phosphoserine and cholesteryl sulfate. 3-Phosphoserine and carcinoembryonic antigen (CEA) in combination can attain a higher accuracy of detection in gastric cancer, plays an inhibitory role against colorectal cancer by inhibiting CDK activity, and cholesterol sulfate was identified as an oncometabolite.^56,57^ In addition, we discovered some less studied metabolites, such as 1-mristoyl-*sn*-glycero-3-phosphocholine, 16b-hydroxyestradiol, d-lyxose, dioctyl phthalate, dl-lactate, Leu-Phe in SCLC, all of which contributed to our diagnostic model for identifying SCLC. The increase in dl-lactate levels, a product of glucose and anaerobic glycolysis, has been reported to promote tumor cell proliferation.^58^ Similarly, intake and uptake of amino acids are boosted to support accelerated cancer cell proliferation due to enhanced consumption in tumors.^59,60^ Further analysis of metabolic pathways revealed that the metabolites in pyruvate metabolism are markedly altered. The alteration may reflect the significant pathological processes during the development and progression of SCLC.

It should be noted that the correlation of the 8 metabolite markers was low. The independence of these biomarkers confirms our finding that they belong to different metabolic pathways, all of which are linked to the development of SCLC. Besides, a mere single biomarker is not powerful enough to discriminate SCLC from NSCLC and healthy participants. The diversity and wide range of our 8-metabolite panel may lend more credibility to the diagnosis of SCLC.

Overall, we established an 8-metabolite panel model that could improve the diagnostic performance for SCLC using metabolomic and lipidomic platforms. Notably, several aspects of our studies exhibited unprecedented novelty. Technically, both ensemble and machine learning methods were carried out to ensure the solidity and convincingness of our diagnostic model. Clinically, our diagnostic model is not limited to stages and clinical variables. Instead, it has value for patients with SCLC at all stages. Although the AUC for SCLC patients in stage I/II was relatively lower than those in stage III and IV, possibly due to the limited number of patients at stage I/II (only 10 patients), it was still identified as a powerful biomarker to discriminate stage I/II SCLC patients from NSCLC patients and healthy controls. Thus, detection of latent patients with SCLC in earlier stages allows early treatment to prolong their survival.

Importantly, our diagnostic model is robust even after adjusting for age, gender, smoking, drinking, and medication history. It would still be interesting to validate our diagnostic model among populations with detailed dietary information to further analyze their impact on the metabolite changes. Furthermore, combining our metabolic panel with NSE and ProGRP could provide a better diagnosis of SCLC with an improved AUC, indicating the complementarity of our diagnostic panel and the conventional biomarkers. Therefore, the notable utilities of our diagnostic biomarker should never be underestimated. The construction of our biomarker panel would facilitate a wide adoption in clinical practice.

The analysis of ctDNA has been adopted for the early detection of cancer, which would ensure an accurate and rapid prediction that can guide treatment strategies. Practically, ctDNA has entered into clinical practice and has been tested in clinical trials. While ctDNA can be reflective of the real-time genomic profiling, the low proportion of ctDNA in circulation is one of the tricky problems that may be encountered. Analysis of metabolomics in the present study, which studies metabolites, also serve as a promising approach for the early detection of cancer. In contrast to ctDNA, which collects genetic information from tumors for early cancer detection, analysis of metabolomics in our study achieves the early detection of cancer by detecting small molecule metabolites. Metabolomics is metabolites-based while ctDNA is based on tumor DNA sequencing. A lower blood volume is needed in metabolomics while a relatively higher blood volume is needed in ctDNA analysis. Moreover, its expense is relatively lower compared with that of ctDNA. Besides, screening tests are needed to identify patients for further screening in metabolomics whereas ctDNA is used as a screening test. Indeed, ctDNA and metabolomics may serve as complementary diagnostic approaches, which may aid early detection of cancers.

However, the limitations of our study should not be neglected. First, our study only included Chinese patients from a single institution. More cohorts with diverse ethnicities from multiple institutions would lend more credibility to the model. Second, the actual value of our model lies in the detection of SCLC before the disease can be diagnosed by imaging and pathology tests, which warrants more extensive prospective trials. Third, diet and lifestyle may affect metabolites; thus, the potential effect of diet and lifestyle on blood metabolites may be underestimated to some extent. Fourth, it would be necessary to include individuals with other rare lung cancers such as pulmonary sarcomatoid carcinoma, and lung neuroendocrine tumor in a separate control group to determine whether these metabolite markers are specific to SCLC alone or whether they are also markers for these rare lung malignancies.

## Conclusion

We have developed a novel and solid diagnostic model for SCLC diagnosis. Our 8-metabolite diagnostic panel offers a feasible and convenient strategy for the diagnosis of SCLC. Our metabolic panel has an excellent diagnostic value, which is informative and meaningful to physicians and can be quickly adopted in clinical practice. Its incorporation in a clinician’s arsenal could significantly improve the ability to identify SCLC patients and even better if combined with the conventional markers NSE and ProGRP. However, further development of prospective clinical trials is warranted to confirm the high precision of our established model for SCLC.

## Supplementary Material

oyad261_suppl_Supplementary_Material

## Data Availability

The data that support the findings of this study are available from the supporting information.
