# Supplementary material for: Construction of a Diagnostic Model for Small Cell Lung Cancer Combining Metabolomics and Integrated Machine Learning
Source: Oncologist. 2023 Sep 14;29(3):e392–401. doi: 10.1093/oncolo/oyad261 (PMC10911920; doi:10.1093/oncolo/oyad261)
Supplement: oyad261_suppl_Supplementary_Material [file oyad261_suppl_supplementary_material.zip › Supplementary Figure Legends.docx]

**Supplementary Figure Legends**

**Supplementary Figure 1: The quality evaluation of metabolites data.** A: The response intensity and retention time of each group in negative/positive ion mode. B: The PCA score plot of each group in negative/positive ion mode.

**Supplementary Figure 2: The quality evaluation of lipidomics data.** A: The number of Peak with RSD in QC samples. B: The PCA score plot of each group.

**Supplementary Figure 3: The lipids difference between the SCLC and control (NSCLC+healthy).** A: three-dimensional PCA score plot of the lipids difference. B: The volcano plots of the lipids difference. C: The PLS-DA scores plot of lipids between SCLC and control. D: The OPLS-DA scores plot of lipids between SCLC and control. E: The bubble chart of lipids between SCLC and control. F: the correlation between lipids

**Supplementary Figure 4: The metabolites difference between the SCLC and control (NSCLC+healthy).** A: The PLS-DA and OPLS-DA scores of metabolites between SCLC and control in negative ion mode. B: The PLS-DA and OPLS-DA scores of metabolites between SCLC and control in positive ion mode.

**Supplementary Figure 5: The weight of metabolites and lipids.**

**Supplementary Figure 6: The correlation of the eight candidate metabolites.**

**Supplementary Figure 7: The function analysis of the eight candidate metabolites.** A: The differential abundance score of the eight candidate metabolites. B: KEGG pathway enrichment analysis of the eight candidate metabolites.

**Supplementary Figure 8: The diagnostic performance of SCLC based on different AJCC stage.** A: The ROC curve of SCLC with stage I/II. B: The clustering heat map in patients with stage I/II. C: The ROC curve of SCLC with stage III. D: The clustering heat map in patients with stage III. E: The ROC curve of SCLC with stage IV. F: The clustering heat map in patients with stage IV.

**Supplementary Figure 9: The diagnostic performance of SCLC based on d-model combined NSE and ProGRP.** A: The diagnostic performance NSE combined d-model. B: The diagnostic performance ProGRP combined d-model. C: The diagnostic performance NSE combined d-model for limited stage SCLC. D: The diagnostic performance ProGRP combined d-model for limited stage SCLC.
