# Supplementary material for: Construction of a Diagnostic Model for Small Cell Lung Cancer Combining Metabolomics and Integrated Machine Learning
Source: Oncologist. 2023 Sep 14;29(3):e392–401. doi: 10.1093/oncolo/oyad261 (PMC10911920; doi:10.1093/oncolo/oyad261)
Supplement: oyad261_suppl_Supplementary_Material [file oyad261_suppl_supplementary_material.zip › Supplementary materials and methods.docx]

**Supplementary materials and methods**

**Extraction of metabolites and lipids**

Metabolites extraction: Serum samples were slowly thawed at 4 ℃. Then, 100 ul sample was added to a precooled methanol/acetonitrile/water solution (2:2:1), vortexed, and the supernatant was incubated at -20 ℃ for 10 min, followed by centrifugation at 14000 × g at 4 ℃ for 20 min. The supernatant was dried under vacuum and redissolved in 100 μL of acetonitrile solution (acetonitrile: water = 1:1) for 15 min. The supernatant was collected for sample injection analysis after centrifugation at 14000 × g at 4 ℃ for 15 min. Finally, 20 µL of supernatant was used for LC–MS/MS analysis.

Lipid extraction: Take an appropriate amount of samples, add 200 μL water and 20 μL internal lipid standard mixture, MP vortex. After that, 800 μL of MTBE was added, samples were vortexed, 240 μL of precooled methanol was added, and samples were vortexed again. The samples were then sonicated for 20 min in a low-temperature water bath and centrifuged (30 min, 14000 × g, 10 ℃). The samples were incubated for 15 min at room temperature, and the upper organic phase was recovered and dried with nitrogen. 200 μL of 90% isopropanol/acetonitrile solution were used to dissolve the sample for mass spectrometry analysis. After vortexing, 90 μL of the compound solution was centrifuged at 14000 × g, 10℃ for 15 min.

**Quality evaluation of experimental data**

Untargeted metabolomics: The same number of samples from the different groups were combined into the quality control (QC) group. The total ion chromatogram of the QC samples was overlapped and compared. The experimental results showed that each color peak's response intensity and retention time overlapped, indicating that the variation caused by the instrument error was slight in the whole experimental process (Supply Figure 1A). In addition, the peaks extracted from all experimental and QC samples were analyzed by unsupervised principal component analysis (PCA). The experimental results showed that the QC samples gather closely in the positive and negative ion modes, indicating that the repeatability of the experiment was good (Supply Figure 1B).

Lipidomics: The same number of samples from the different groups were mixed into the QC sample. The smaller the relative standard deviation (RSD) of the ion peak abundance of the QC sample indicated excellent stability of the instrument, reflecting the quality of the data. In this experiment, the number of peaks with RSD ≤ 30% in QC samples accounted for more than 80% of the total peak number of QC samples, indicating that the stability of the instrument analysis system was good (Supply Figure 2A). The ion peaks extracted from all experimental samples and QC samples were analyzed by PCA after Pareto-scaling. The experimental results showed that the QC samples were closely gathered together, indicating that the repeatability of the experiment was good (Supply Figure 2B).

**LC-MS/MS analysis**

Untargeted metabolomics: The samples were separated using an Agilent 1290 Infinity LC ultra-high-performance liquid chromatography (UHPLC) HILIC column. During the whole analysis process, the sample was placed on an automatic sampler at 4 ℃. In order to avoid the influence caused by the fluctuation of the instrument detection signal, a random sequence was used for continuous analysis of the samples. QC samples were inserted into the sample queue to monitor and evaluate the system's stability and the experimental data's reliability. The primary and secondary spectra of the samples were collected using AB Triple TOF 6600 mass spectrometers.

Lipidomics: The samples were separated using the UHPLC Nexera LC-30A ultra-high-performance liquid chromatography system. During the whole analysis process, the sample was placed on an automatic sampler at 10 ℃. In order to avoid the influence caused by the fluctuation of the instrument detection signal, a random sequence was adopted to carry out the continuous analysis of the samples. The positive and negative ion modes of electrospray ionization (ESI) were detected. The samples were separated by UHPLC and analyzed by mass spectrometry with Q Exactive series mass spectrometer (Thermo Scientific^TM^). The mass-charge ratio of lipid molecules to lipid fragments was collected according to the following methods: 10 fragment profiles (MS^2^ scan, HCD) were collected after each full scan. LipidSearch was used to identify lipids and standard internal lipids for peak recognition, peak extraction, lipid identification (secondary identification), etc. The main parameters were precursor tolerance 5 ppm, product tolerance 5 ppm, and product ion threshold 5%. The quality of the data extracted by LipidSearch was evaluated, and then the data was analyzed.
