# Supplementary material for: Construction of a Diagnostic Model for Small Cell Lung Cancer Combining Metabolomics and Integrated Machine Learning
Source: Oncologist. 2023 Sep 14;29(3):e392–401. doi: 10.1093/oncolo/oyad261 (PMC10911920; doi:10.1093/oncolo/oyad261)
Supplement: oyad261_suppl_Supplementary_Material [file oyad261_suppl_supplementary_material.zip › Supplementary Table 1.docx]

**Supplementary Table 1. The clinical characteristics for participants in the study**

| **Variables** | **Total (n = 461)** | | |
| --- | --- | --- | --- |
|  | **SCLC (n = 191)** | **NSCLC (n = 173)** | **Healthy (n = 97)** |
| **Age, years** |  |  |  |
| <65 | 115 | 89 | 95 |
| ≥65 | 76 | 84 | 2 |
| **Sex** |  |  |  |
| Female | 56 | 56 | 45 |
| Male | 135 | 117 | 52 |
| **Smoking** |  |  | n.a. |
| Yes | 111 | 80 |  |
| No | 80 | 93 |  |
| **Drinking** |  |  | n.a. |
| Yes | 70 | 51 |  |
| No | 121 | 122 |  |
| **Medication history** |  |  | n.a. |
| Yes | 52 | 63 |  |
| No | 139 | 110 |  |
| **Histology** |  |  | n.a. |
| SCLC | 191 |  |  |
| squamous |  | 82 |  |
| adenocarcinoma |  | 91 |  |
| **Stage** |  | n.a. | n.a. |
| Limited stage | 87 |  |  |
| Extensive stage | 104 |  |  |
| **NSE (ng/ml)** |  |  | n.a. |
| Median | 41.47 | 14.515 |  |
| Range | 3.4-740 | 2.9-95.87 |  |
| **ProGRP (pg/ml)** |  |  | n.a. |
| Median | 1951 | 47.8 |  |
| Range | 10.7-39040 | 3-85 |  |

SCLC: small cell lung cancer; NS: non-small cell lung cancer; NSE: neuron-specific enolase; ProGRP: Pro-gastrin-releasing peptide; n.a.: not available.
