# Supplementary material for: Construction of a Diagnostic Model for Small Cell Lung Cancer Combining Metabolomics and Integrated Machine Learning
Source: Oncologist. 2023 Sep 14;29(3):e392–401. doi: 10.1093/oncolo/oyad261 (PMC10911920; doi:10.1093/oncolo/oyad261)
Supplement: oyad261_suppl_Supplementary_Material [file oyad261_suppl_supplementary_material.zip › Supplementary Table 6.docx]

| Variables | AUC | 95% CI |
| --- | --- | --- |
| Age, years |  |  |
| <65 | 0.935 | 0.909-0.961 |
| ≥65 | 0.925 | 0.892-0.958 |
| Gender |  |  |
| Female | 0.917 | 0.876-0.959 |
| Male | 0.937 | 0.912-0.963 |
| Smoking |  |  |
| Yes | 0.937 | 0.914-0.960 |
| No | 0.922 | 0.888-0.956 |
| Drinking |  |  |
| Yes | 0.924 | 0.887-0.961 |
| No | 0.934 | 0.909-0.959 |
| Medication history |  |  |
| Yes | 0.926 | 0.886-0.967 |
| No | 0.933 | 0.909-0.957 |

**Supplementary Table 6: The AUC of d-model based on different clinical variables**
