# Supplementary figures and images for: Construction of a Diagnostic Model for Small Cell Lung Cancer Combining Metabolomics and Integrated Machine Learning
Source: Oncologist. 2023 Sep 14;29(3):e392–401. doi: 10.1093/oncolo/oyad261 (PMC10911920; doi:10.1093/oncolo/oyad261)

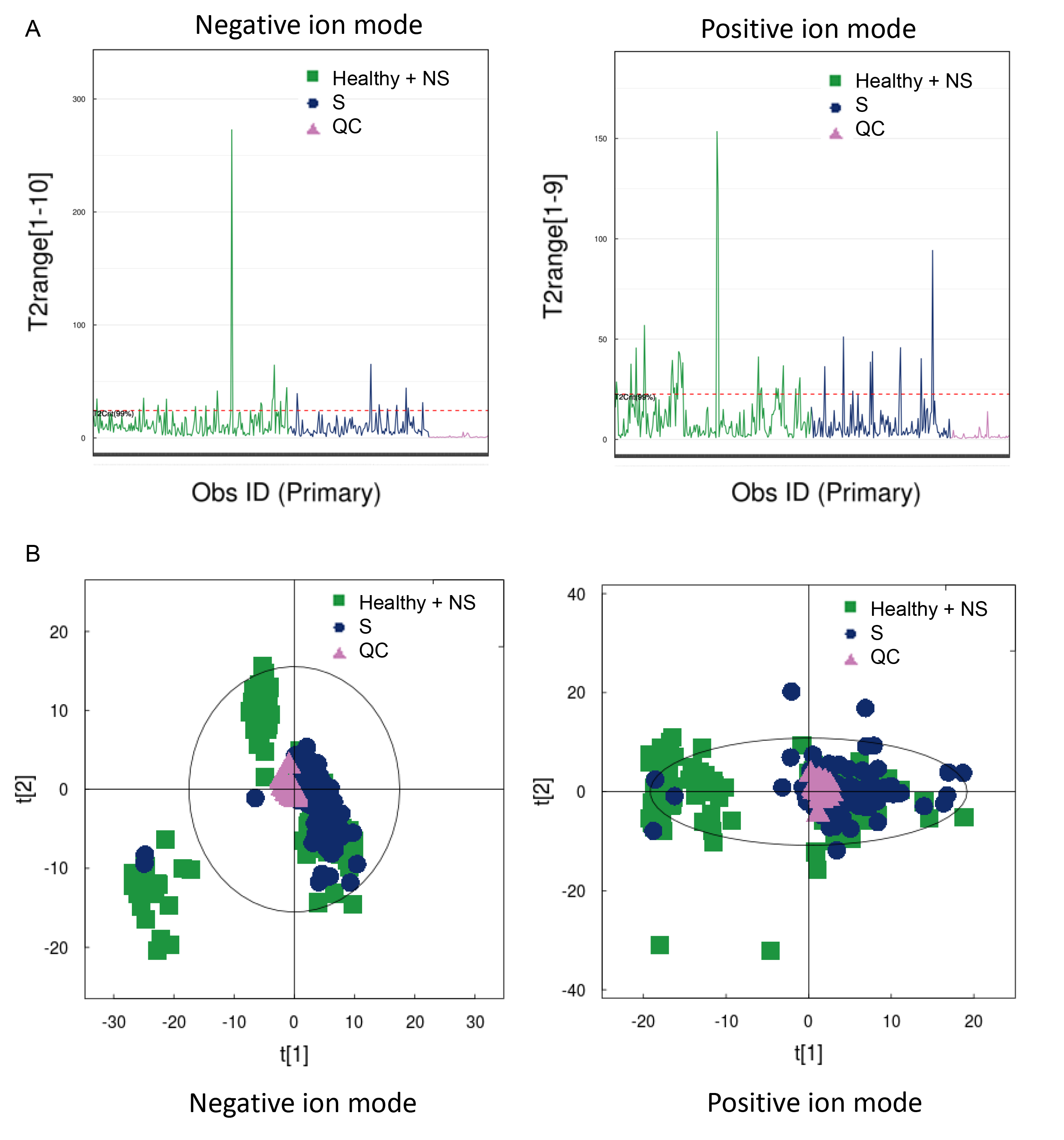

Supplement: oyad261_suppl_Supplementary_Material [file oyad261_suppl_supplementary_material.zip › Supply figure 1.tif]

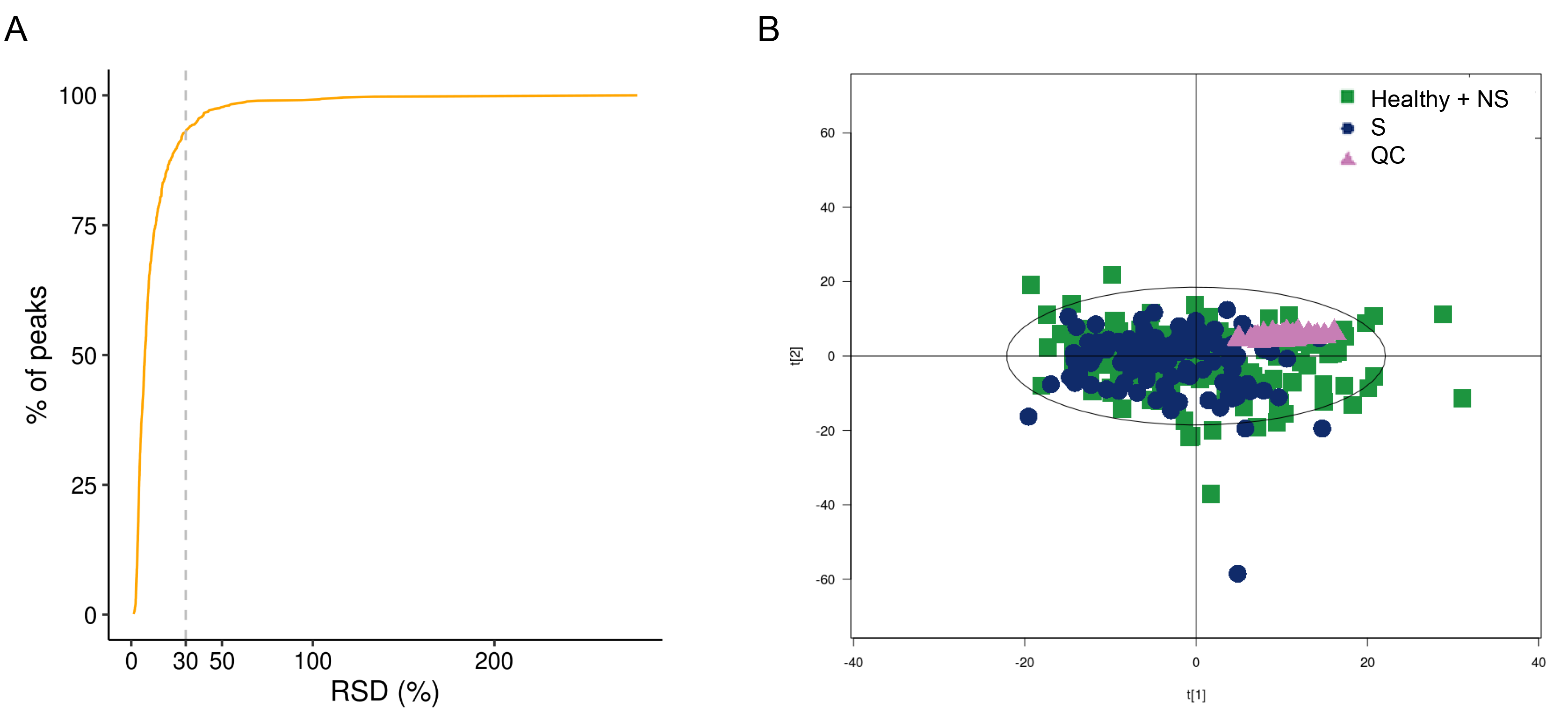

Supplement: oyad261_suppl_Supplementary_Material [file oyad261_suppl_supplementary_material.zip › Supply figure 2.tif]

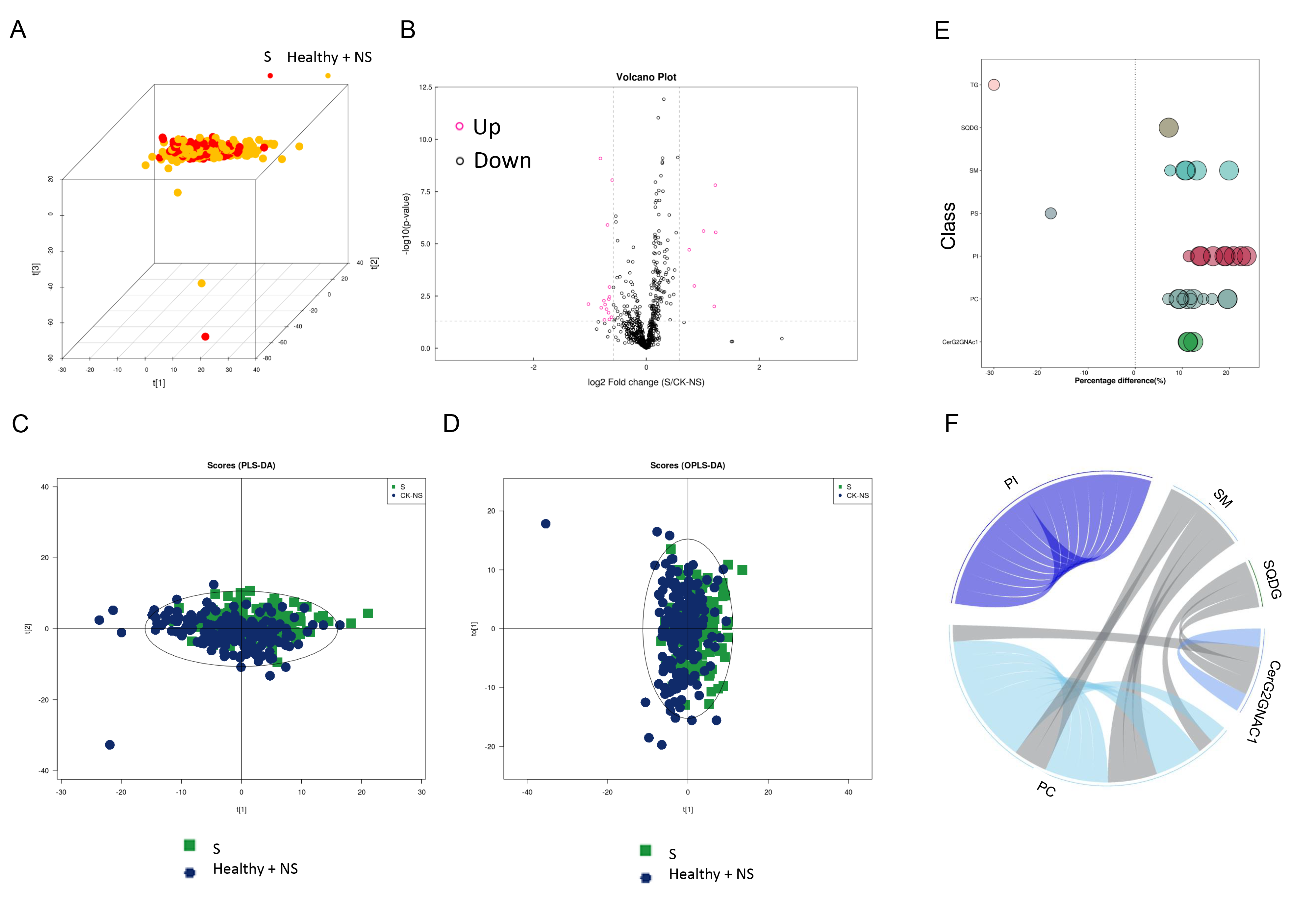

Supplement: oyad261_suppl_Supplementary_Material [file oyad261_suppl_supplementary_material.zip › Supply figure 3.tif]

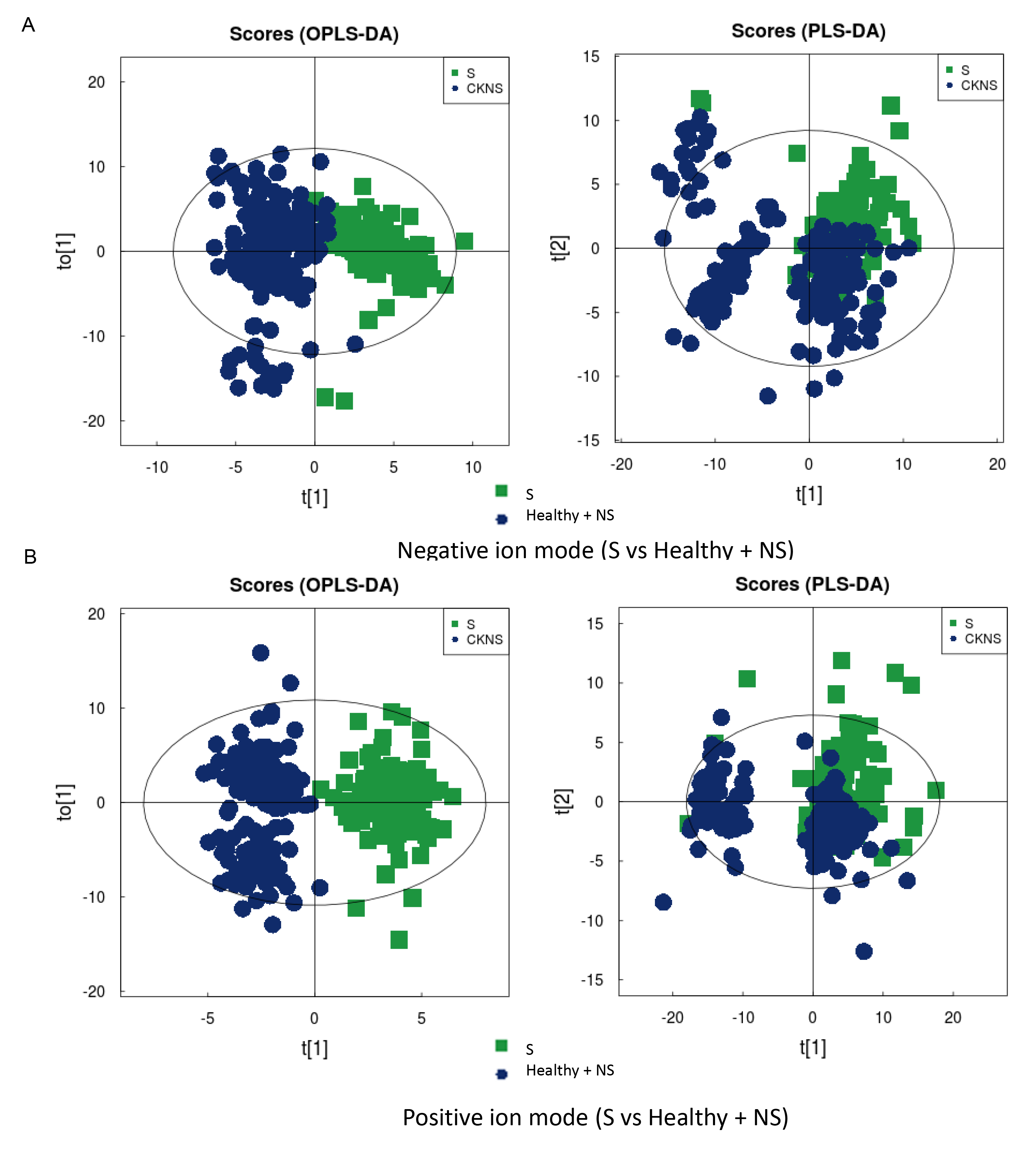

Supplement: oyad261_suppl_Supplementary_Material [file oyad261_suppl_supplementary_material.zip › Supply figure 4.tif]

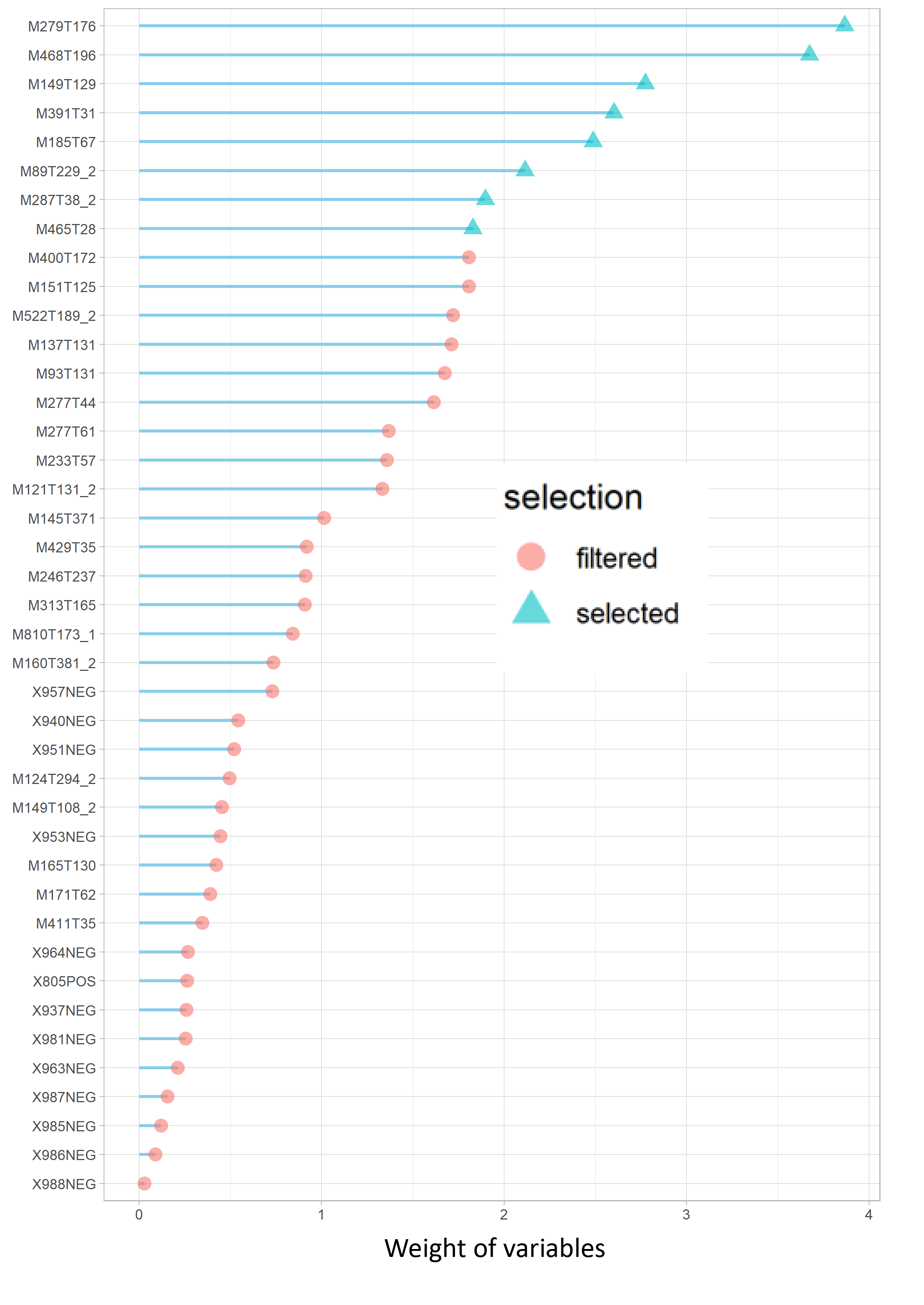

Supplement: oyad261_suppl_Supplementary_Material [file oyad261_suppl_supplementary_material.zip › Supply figure 5.tif]

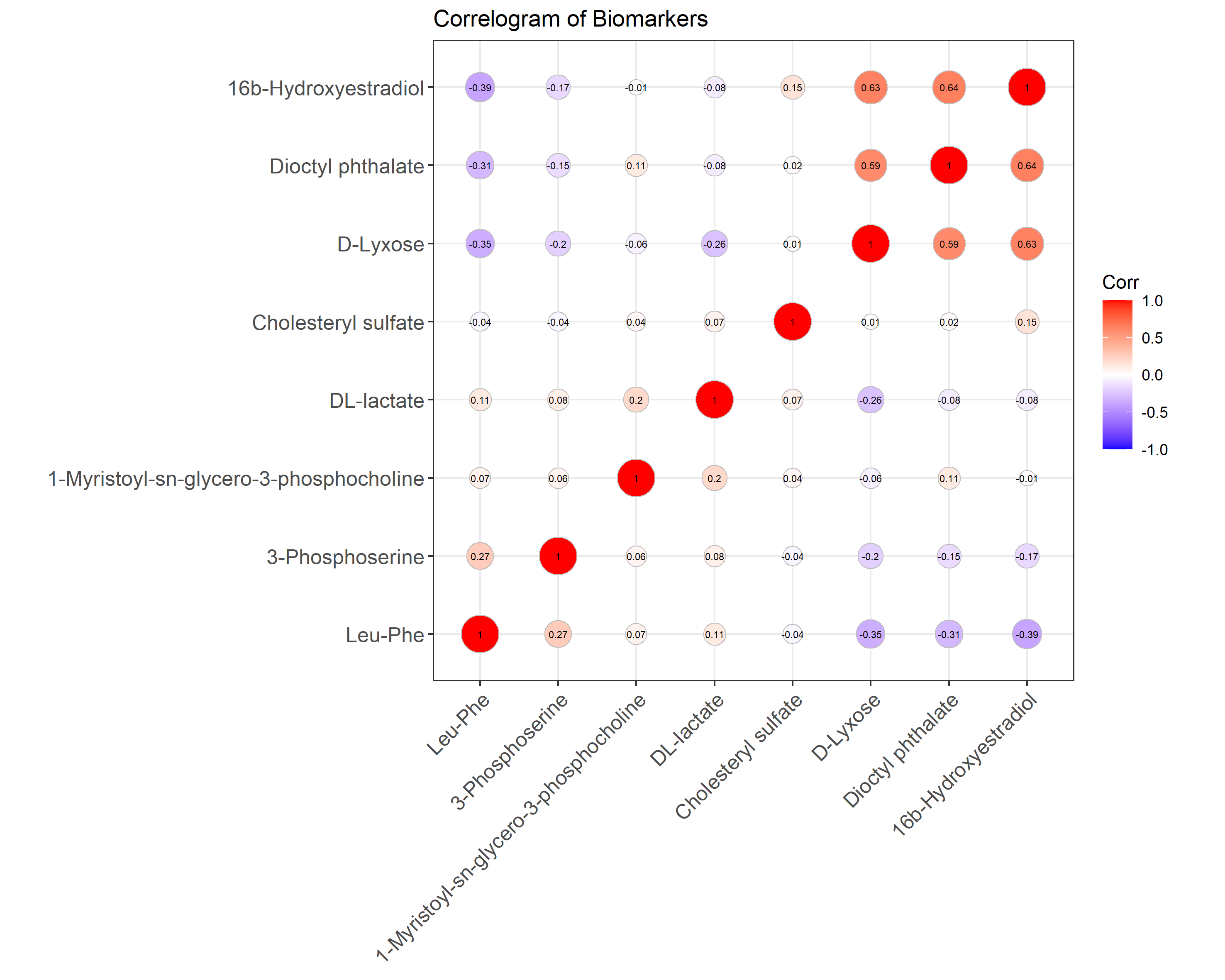

Supplement: oyad261_suppl_Supplementary_Material [file oyad261_suppl_supplementary_material.zip › Supply figure 6.tif]

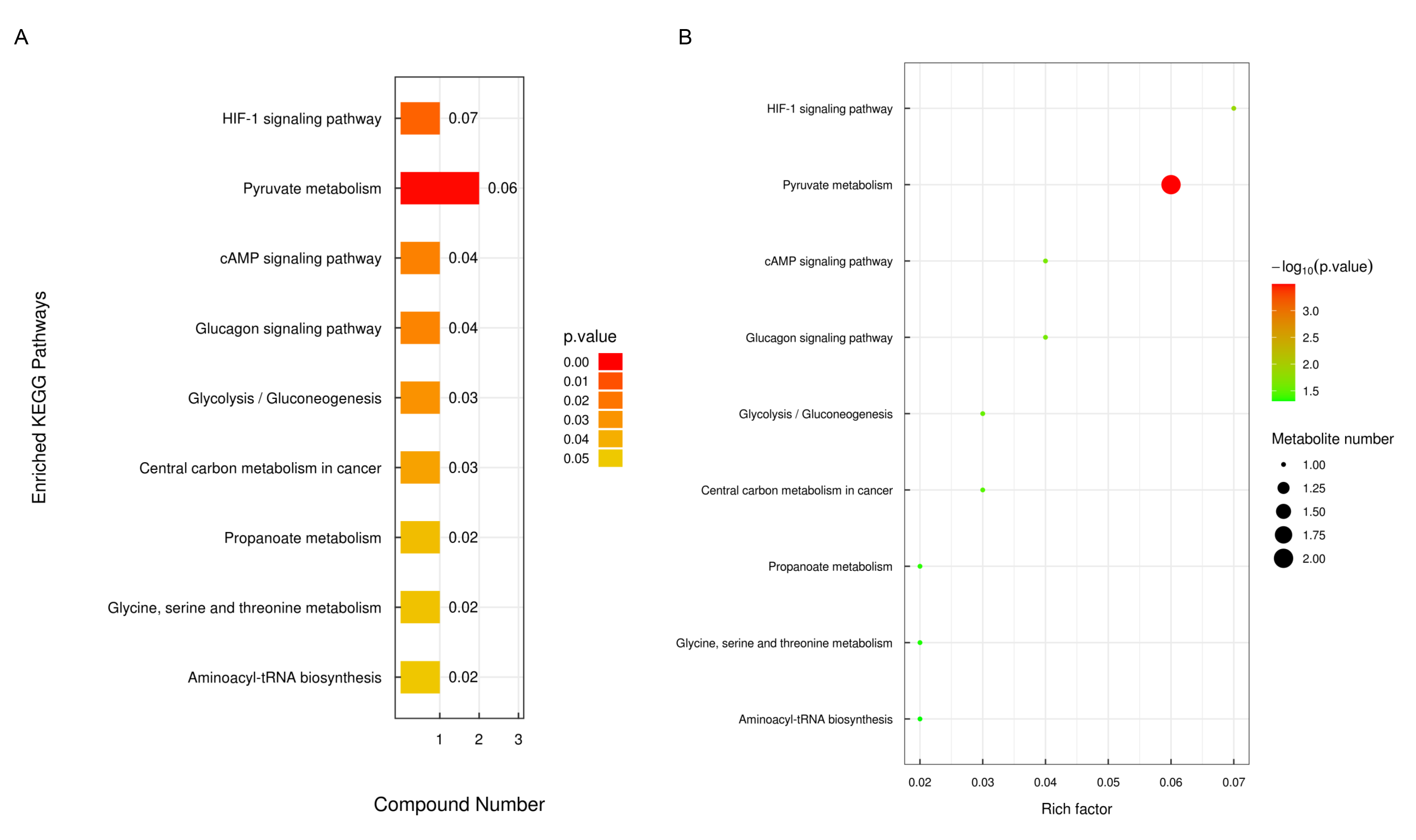

Supplement: oyad261_suppl_Supplementary_Material [file oyad261_suppl_supplementary_material.zip › Supply figure 7.tif]

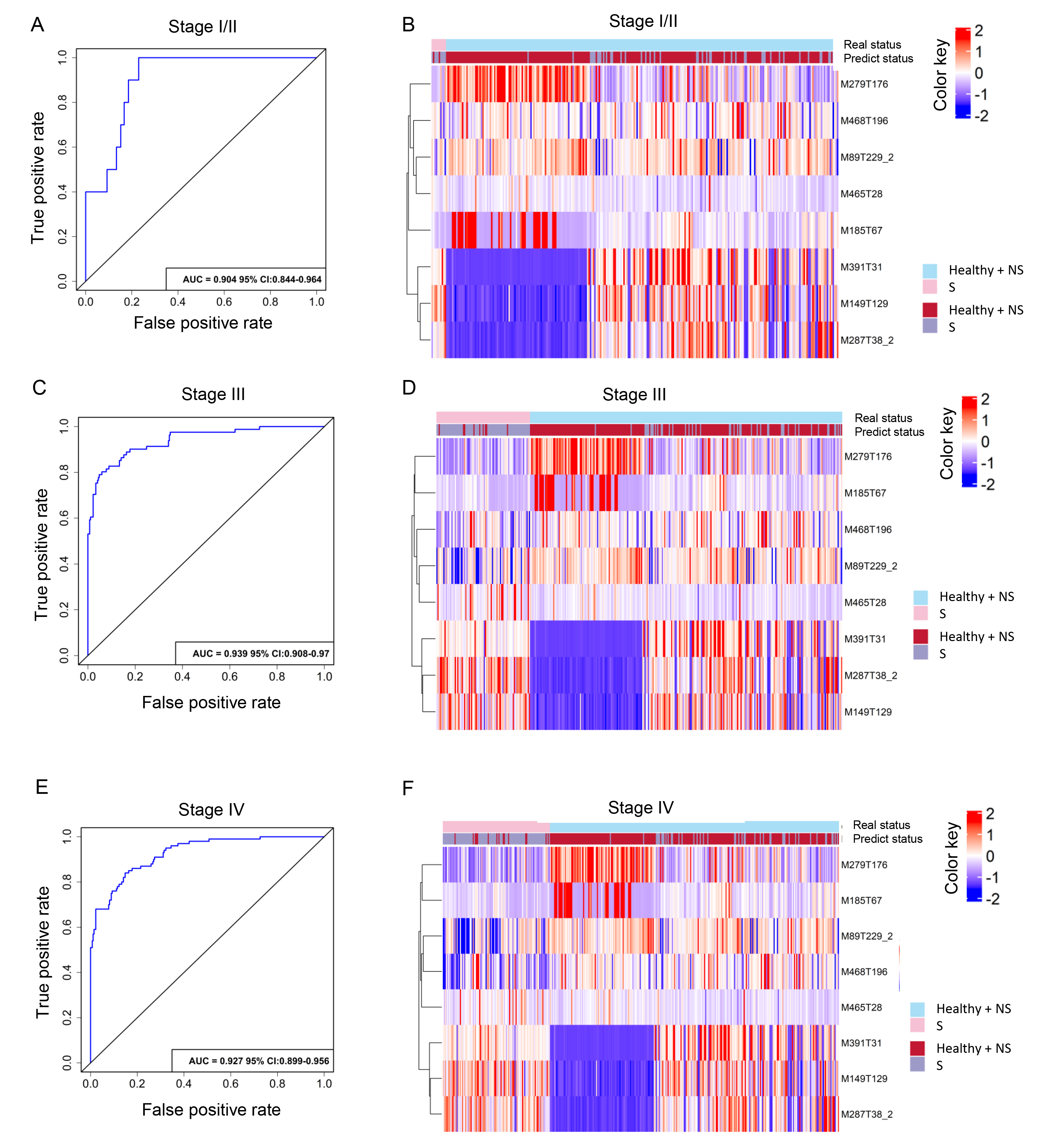

Supplement: oyad261_suppl_Supplementary_Material [file oyad261_suppl_supplementary_material.zip › Supply figure 8.tif]

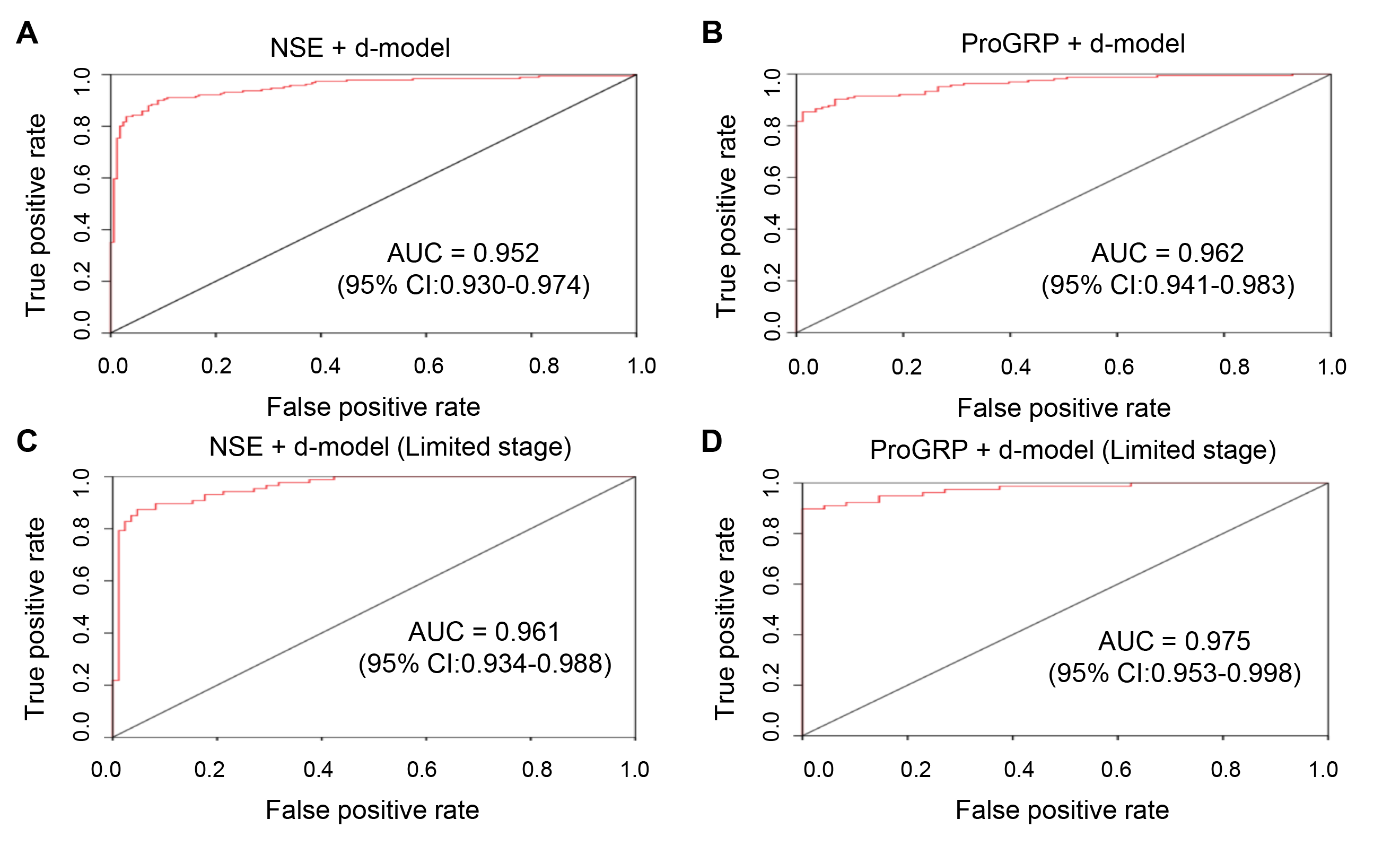

Supplement: oyad261_suppl_Supplementary_Material [file oyad261_suppl_supplementary_material.zip › Supply figure 9.tif]
